# Hitherto Unrecognized Fluorescence Properties of Coniferyl Alcohol

**DOI:** 10.3390/molecules15031645

**Published:** 2010-03-11

**Authors:** Komandoor Elayavalli Achyuthan, Paul David Adams, Supratim Datta, Blake Alexander Simmons, Anup Kumar Singh

**Affiliations:** 1 Joint BioEnergy Institute (JBEI), Emeryville, CA 94550, USA; 2 Biosensors and Nanomaterials Department, Sandia National Laboratories, Albuquerque, NM 87185, USA; 3 Lawrence Berkeley National Laboratory, Berkeley, CA 94720, USA; E-Mail: pdadams@lbl.gov (P.D.A.); 4 Sandia National Laboratories, Livermore, CA 94550, USA; E-Mails: basimmo@sandia.gov (B.A.S.); sdatta2@lbl.gov (S.D.); aksingh@sandia.gov (A.K.S.)

**Keywords:** coniferyl alcohol, fluorescence, laccase, peroxidase, high throughput screening, lignin, biofuels

## Abstract

We instituted a quasi-quality assurance program for demonstrating coniferyl alcohol’s fluorescence and fluorescence diminishment following enzymatic oxidation. The magnitude of diminishment was a measure of catalysis. High throughput screening was performed in *pseudo*-kinetic and endpoint modes by measuring the fluorescence at 416 nm following excitation at 290, 310 or 340 nm. Dose-response tracings were linear between two and three orders of magnitude with average limits of detection and quantitation of 1.8 and 6.9 μM coniferyl alcohol, respectively. Oxidation was evident with 0.025 μg/mL laccase or 0.003 μg/mL peroxidase or inside 5 min using 0.5 μg/mL laccase or 5 μM substrate. Sodium chloride inhibited (IC_50_, 25 mM) laccase oxidation of coniferyl alcohol. Fluorescence from 10 concentrations (1 to 1000 μM) of coniferyl alcohol was stable for 24 hours over 14 excitation/emission cycles at 3 different combinations of excitation and emission wavelengths. In conclusion, coniferyl alcohol absorption and fluorescence assays should facilitate biomass lignin analyses and improve delignification.

## 1. Introduction

Lignin is composed of three main phytochemicals: the monolignols of coniferyl alcohol (CA), sinapyl alcohol (SA) and *p*-coumaryl alcohol (*p*-CA), that are cross-linked through β-O-4, β-1, β-5, β-β, 4-O-5 and 5-5 bonds [1,2]. Depending on the plant species or tissue type, the monolignol content varies. Guaiacyl lignin of softwood (Gymnosperms), comprised principally of CA, is more resistant to delignification, whereas guaiacyl-syringyl lignin of hardwood (Angiosperms) containing CA and SA is easily degraded. Graminaceous lignin (grass) has high levels of *p*-CA [2]. Lignin composition is thus causally related to biomass recalcitrance, impeding the development of cost-effective biofuels [1,2,3]. Delignifying enzymes have not been identified in plants. However, filamentous white and brown rot fungi (*Ascomycetes* and *Basidomycetes*) degrade hardwood and softwood through phenoloxidative enzymes such as laccase and peroxidase [2]. For example, the white rot *Basidomycetes Trametes versicolor* degrades lignin through oxidative, demethoxylating and demethylating activities or *via* redox shuttle mediators (RSM) [2,4,5]. 

Knowledge of monolignol composition will allow genetic modifications for changing the lignin content towards reducing recalcitrance and enhancing saccharification [1,2]. Monolignol estimation might facilitate better pre-treatment strategies to enhance delignification [3]. We recently surveyed the methods for analyzing CA [6] and described high throughput screening (HTS) assays [7] for CA and *p*-cresol (both potential RSMs) [6,8,9]. Under a quasi-quality assurance (QA) program, we now describe hitherto unrecognized fluorescence properties of CA. We also provide a non-specialist primer on fluorescence in order to contextualize our findings. Our HTS assays utilized fluorescence changes due to laccase and peroxidase catalysis for detecting and quantitating CA oxidation. Our fluorescent assays are operable under *pseudo*-kinetic and endpoint modes with optical “turn off” signaling.

## 2. Results and Discussion

### 2.1. CA Quality Assurance Program

To our best information, the fluorescence properties of CA have not been described. We therefore instituted a quasi-QA program to demonstrate CA’s fluorescence. We purchased CA from three independent vendors. Two of the sources were in geographically separate locations in the United States and the third was an overseas vendor. Absorption and fluorescence data were collected by two investigators, working independently at two different test sites, using three separate microplate spectrometers and two different enzymes (laccase and peroxidase) which used CA as the substrate. Similar experiments were carried out in two microwell density formats (96 or 384) using two different microplates (UV-transparent or white). The last metric was essential due to the potential for interference from the white plate’s intrinsic fluorescence [10]. Tests conducted several weeks apart yielded similar profiles testifying to the reproducibility of the fluorescence properties of CA. We employed the well known absorption assays [6] (references cited therein) in order to uncover any differences amongst the CA samples from the three vendors before describing the fluorescence assays.

It is well known that CA absorption at 260 nm decreases following enzymatic oxidation [6 and references cited therein]. For the same 500 μM concentration, in the absence of laccase, CA from Sigma-Aldrich had the highest absorbance in pH 4.5 buffer (2.681 ± 0.069 A.U), followed by CA from Acros Organics (1.520 ± 0.024), whilst CA from Alfa Aesar had the least absorbance (0.675 ± 0.004) (Figure 1A). The CA samples from Acros Organics and Alfa Aesar were rapidly oxidized with saturation between 0.25 to 1.0 μg/mL enzyme. On the other hand, CA from Sigma-Aldrich underwent gradual and linear oxidation over the 20 min reaction period with no evidence of saturation even at ~1.0 μg/mL laccase. However, the saturation of the reaction velocity under acidic conditions is difficult to estimate due to the formation of light-scattering dehydrogenation polymers (DHPs) that transformed the reaction mixtures into cloudy suspensions [6]. 

When laccase oxidation of CA samples was examined under basic pH conditions in endpoint mode at 60 min using the previously established 290 nm absorption wavelength [6], similar features were observed (Figure 1B). The DHPs appeared to dissolve entirely in base resulting in the reactions becoming clear and allowing the estimation of reaction velocity. In the absence of laccase, 500 μM of CA from Sigma-Aldrich once again had the highest absorption in base (3.227 ± 0.050 A.U.) followed by CA from Acros Organics (2.056 ± 0.039 A.U.) and CA from Alfa Aesar trailing at 1.252 ± 0.006 A.U. As reported previously [6], CA absorption in pH 4.5 buffer was lower than its absorption in base, regardless of the source. Similar to pH 4.5 reactions, the CA from Sigma-Aldrich underwent a gradual oxidation compared to CA from Acros Organics or Alfa Aesar in endpoint mode (Figure 1B). The oxidation of CA in the 60 min endpoint reactions with 1.0 μg/mL laccase could be rank ordered as Sigma-Aldrich (58%) > Acros Organics (43%) > Alfa Aesar (18%).

**Figure 2:**
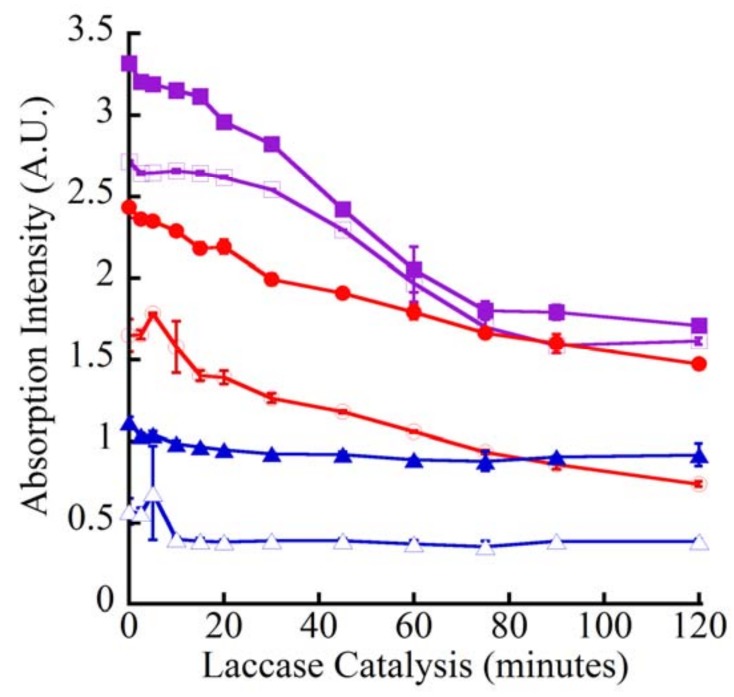
Laccase kinetic (260 nm) and *pseudo-*kinetic (290 nm) oxidation of CA. Laccase (0.1 μg/mL) was used to oxidize 500 μM CA in 100 μL of pH 4.5 buffer using UV-transparent 96-well microplate. Absorbance decrease from identical reactions was measured at 260 nm (open symbols) for the indicated time periods. *Pseudo*-kinetic reactions were stopped with base (100 mM NaOH) and absorbance decreases were measured at 290 nm (closed symbols). The CA samples analyzed were from Alfa Aesar (open and closed triangles, blue tracings), Acros Organics (open and closed circles, red tracings) and Sigma-Aldrich (open and closed squares, purple tracings).

Kinetics of laccase-catalyzed oxidation of CA were executed in *pseudo*-kinetic mode with 0.1 μg/mL laccase and interrogated using 260 nm (pH 4.5) and 290 nm (base) absorption wavelengths. The absorbance values of the Sigma-Aldrich, Acros Organics and Alfa Aesar CA samples in pH 4.5 buffer (2.713 ± 0.007 A.U., 1.649 ± 0.100 A.U. and 0.569 ± 0.080 A.U.) and in base (3.317 ± 0.004 A.U., 2.434 ± 0.066 A.U. and 1.113 ± 0.035 A.U.) were similar to earlier values (Figure 1) confirming reproducibility. Reaction progress of Sigma-Aldrich and Acros Organics CA samples was discernible between 5 and 90 min with 0.1 μg/mL laccase. Alfa Aesar CA showed the least substrate activity in both *pseudo*-kinetic (260 nm) and endpoint (290 nm) absorption assays (Figure 2). However, the oxidation profiles of all CA samples were similar regardless of the interrogation wavelength or the pH. 

The absorption assays demonstrated that all three CA samples were oxidized by laccase to different extents depending on the source, with Sigma-Aldrich CA exhibiting the highest molar absorptivity and the most clearly delineated reaction kinetics with laccase. It would be speculation regarding the reasons for the differences between the CA samples and might be attributed to trace impurities in the CA from Alfa Aesar and to a lesser extent in the CA from Acros Organics. These contaminants might be quenching the absorption of CA and/or inhibiting the activity of the laccase. Interestingly, following basification of the laccase reactions, the mixtures containing CA from Acros Organics and Alfa Aesar turned pale yellow whereas similar reactions using Sigma-Aldrich CA remained colorless. The absorption assays showed that whereas all three CA samples could be used for investigating their fluorescence properties, the CA from Sigma-Aldrich might be the best choice. Therefore, during initial experiments we tested CA from all three vendors as part of our QA program, but later focused upon the Sigma-Aldrich CA for detailed fluorescence analyses.

### 2.2. CA Absorption Spectra and Overlap with Excitation Spectra

**Figure 3:**
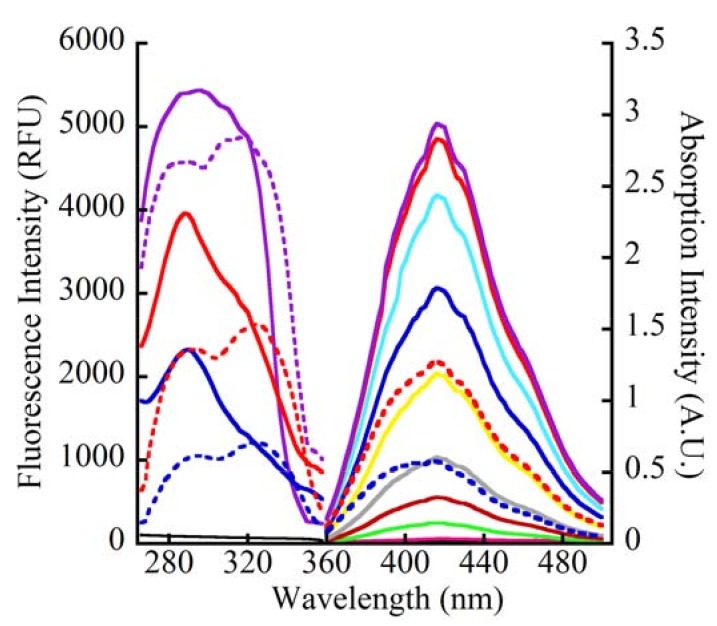
Absorption, excitation and emission spectra of CA. Left panel: Unmodified CA (100 μL of 500 μM CA in 100 mM NaOH) was dispensed into a 96-well UV-transparent microplate. Absorption was monitored between 250 to 350 nm in 2 nm intervals (solid tracings) for CA purchased from Alfa Aesar (blue), Acros Organics (red) and Sigma-Aldrich (purple). Excitation spectral profiles (broken tracings) were monitored between 250 to 350 nm in 2 nm intervals (emission = 420 nm) from CA purchased from Alfa Aesar (blue), Acros Organics (red) and Sigma-Aldrich (purple). The absorption and excitation profiles from 100 mM NaOH background are shown by the solid black tracing close to the abscissa. Right panel: The emission profiles of 100 μL of CA in 100 mM NaOH ranged from 360 to 500 nm in 2 nm intervals with an excitation wavelength of 290 nm. Fluorescence emission with increasing intensities represented by the solid tracings were from the following concentrations (μM) of Sigma-Aldrich CA: 1.0 (magenta), 2.5 (green), 5 (maroon), 10 (grey), 25 (yellow), 50 (blue), 100 (aqua), 250 (red) and 500 (purple). The broken red and blue tracings represent the fluorescence emission from 500 μM of CA from Acros Organics and Alfa Aesar, respectively. The black tracing close the abscissa is the background from 100 mM NaOH.

The absorption profiles of CA from Acros Organics and Alfa Aesar were similar to the absorption profile of Sigma-Aldrich CA (Figure 3), although as noted above their absorption intensities declined as follows: Sigma-Aldrich > Acros Organics > Alfa Aesar. The absorption maximum for all three CA samples was 290 nm. All three absorption profiles spanned 250 to 350 nm. The CA from Sigma-Aldrich had a broader peak compared to the sharper peaks of CA samples from Acros Oganics and Alfa Aesar. The Sigma-Aldrich CA absorption declined steeply resulting in a bell shaped curve (Figure 3, left panel, solid purple tracing). The declining profiles of CA from Acros Organics and Alfa Aesar were more gradual resulting in a squat shoulder, bathochromic to the absorption maximum (Figure 3, left panel, solid red and solid blue tracings). 

**Figure 4:**
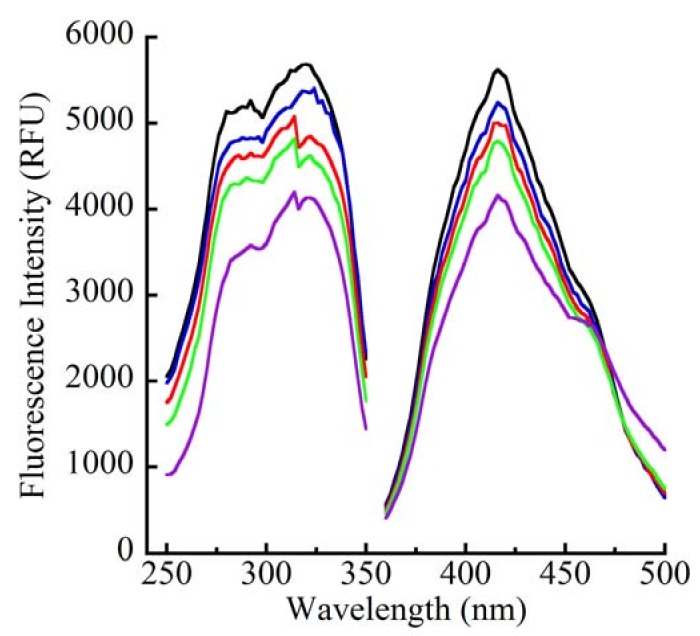
Excitation and emission spectra of laccase-oxidized CA. Increasing concentrations of laccase were reacted with 250 μM CA in 100 μL of citrate buffer, pH 4.5. Reactions were carried out in 96-well UV-transparent microplate and stopped at 30 min through the addition of 250 mM KOH. The pH 4.5 reactions were cloudy due to the formation of DHPs. The cloudiness disappeared upon basification yielding clear solutions that were scanned over the excitation wavelengths of 250 to 350 nm in 2 nm intervals (λem, 416 nm) (left panel). The black tracing with the highest fluorescence is the excitation spectrum of 250 μM unmodified CA. The profiles with decreasing fluorescence intensities represent the excitation spectra of CA oxidized by 0.01 (blue), 0.05 (red), 0.1 (green) and 0.25 (purple) μg/mL laccase. Samples were then excited using 310 nm wavelength and the emission was scanned between 360 and 500 nm in 2 nm intervals (right panel). The black tracing with the highest fluorescence intensity is the emission spectrum of 250 μM unmodified CA. The tracings with progressively declining fluorescence intensities represent the emission spectra of CA oxidized using 0.01 (blue), 0.05 (red), 0.1 (green) and 0.25 (purple) μg/mL laccase.

We next scanned for CA excitation over the same wavelength range as its absorption spectrum and measured the emission at 420 nm, thereby generating an excitation spectrum (Figure 3, left panel, broken tracings). Each CA’s excitation peak was broad spanning 60 nm between 275 and 335 nm. The excitation profiles of CA from the three vendors were nearly identical including the characteristic double excitation maxima composed of a smaller peak between 278 to 296 nm and a slightly larger peak at 304 to 324 nm. The twin peaks were observed in multiple experiments over several days, in experiments conducted by different investigators at different locations, and were also observed in the excitation spectra of laccase-oxidized CA (Figure 4). Significant (~80% of maximum) emission was noted between 280 to 330 nm excitation wavelengths (Figure 3). Similar to the absorption profiles, the three CA samples showed marked differences in their emission intensities declining as follows: Sigma-Aldrich > Acros Organics > Alfa Aesar. These results offered the first evidence for the fluorescence properties of CA and supported our QA model.

The overlapping profiles of the absorption and the excitation spectra (250 to 350 nm) for all three CA samples were as expected. For aromatic compounds or compounds containing unsaturated bonds, the excitation spectrum is usually similar or identical to the absorption spectrum [11]. If we assume the quantum efficiency of fluorescence is independent of the excitation energy, then the signal intensity of the excitation spectrum is an indication of the variation in the photons absorbing energy as a function of the wavelength. Such was the case with the dilute 500 μM solution of CA that was used to generate the absorption and excitation spectra. Therefore, the fraction of the exciting energy absorbed by CA is proportional to its absorption coefficient. Consequently, the excitation and absorption spectra of CA were overlapping (Figure 3). Spectral overlap also suggested the presence of a homogenous CA chromophore/fluorophore in solution. Although the excitation spectrum is often used to analyze the fluorescence of a mixture of absorbing compounds, in this case, the spectra allowed us to document the fluorescence properties of CA. Fluorescence assays are amongst the most widely used biochemical analysis tools and are regarded as the "gold standard" for detecting analytes [12].

### 2.3. Emission and Excitation Spectra of CA

The emission spectral profiles of CA samples from the three commercial sources were also nearly identical to one another (Figure 3, right panel). Consistent with the excitation spectral data, the CA from Acros Organics had lower emission intensity compared to Sigma-Aldrich CA but was higher than the fluorescence of CA from Alfa Aesar. Increasing concentrations of Sigma-Aldrich CA yielded progressively increasing fluorescence emission intensities between 1 μM and 250 μM; at 500 μM CA, the dose-response became negligible. The emission profile had maximum fluorescence intensity between 416 to 420 nm for all CA samples, a >100 nm Stokes shift, that avoided Rayleigh scattering (Figure 3). Nearly 75% of maximum RFU was observed between the emission wavelengths of 412 nm to 446 nm with all samples. The 500 μM Acros Organics CA had an emission profile overlapping the 25 μM emission profile of CA from Sigma-Aldrich. Likewise, the emission profile of 500 μM CA from Alfa Aesar was coincident with the emission profile of 10 μM CA from Sigma-Aldrich. However, this does not mean that Sigma-Aldrich CA is 20- and 50-times more fluorescent on a molar basis compared to CA samples from Acros Organics and Alfa Aesar, respectively. It is clear from Figure 3 that the fluorescence intensity ceased to track linearly with CA concentration at 500 μM. 

We empirically determined the excitation maximum by generating excitation spectra from 260 to 350 nm in 10 nm intervals (420 nm emission). Similar excitation maxima and excitation profiles were obtained using 96-well UV-transparent and white microplates by testing nine concentrations of Sigma-Aldrich CA (1 to 500 μM). Through these analyses, we concluded that 290 to 320 nm was the excitation maximum for CA. At flanking wavelengths, the emission intensity declined. For example, using 96-well white microplate and 270 nm excitation, the fluorescence of 250 μM Sigma-Aldrich CA was 5837 RFU compared to 7819 RFU at 310 nm (25.3% drop). Likewise, at the excitation wavelength of 330 nm, the fluorescence was 6771 RFU (13.4% drop). Further out, the CA fluorescence dropped more steeply. At 260 nm excitation, the emission was 4353 RFU (55.7% of λmax) and at 350 nm it was 2622 RFU (33.5% of λmax). The higher fluorescence intensity of CA in white plate compared to the UV-transparent clear plate was due to light reflection from the white well walls. The data from the three samples supported our QA model and confirmed the fluorescence of CA. 

The emission spectrum of CA, similar to most fluorophors, was red-shifted with respect to the absorption and excitation spectra. The excitation and emission spectra represent the probability distribution functions that a photon with particular quantum energy will be absorbed by CA. The excited state of CA loses the absorbed energy through the release of a second photon as fluorescence emission with lower energy and longer wavelength. This is because the fluorescence of CA is derived from the lowest vibrational level (*v* = 0) of the lowest electronically excited singlet state (S). The absorption of high-energy photons by CA is accompanied by energy loss through non-radiative processes before emission occurs. Consequently, the emitted photons have less energy compared to the absorbed photons, and the fluorescence is red-shifted relative to the absorption and excitation spectra (Figure 3). In compliance with Kasha's rule [11], CA displayed the same emission profiles irrespective of the excitation energy (λ), although the emission intensity varied over these wavelengths (Figure 3). The identical emission spectra at different excitation wavelengths may be explained as follows. Upon excitation, CA enters a higher electronic and vibrational level and the excess energy is lost over pico- to nano-second (10^-12^ to 10^-9^) lifetimes. CA drops to the lowest vibrational level of the first excited state (S1), from where emission takes place [11]. Due to the rapid relaxation lifetimes, the emission spectra of CA were identical at various excitation wavelengths and independent of the excitation energy. CA in solution does not display the detailed vibrational structure, resulting in the emission spectrum appearing as a broad band. 

### 2.4. Base Optimization

The CA fluorescence was observed optimally under basic pH conditions. Either 100 mM NaOH or 250 mM KOH resulted in maximum fluorescence and S/B. For example, with 10 μM of Sigma-Aldrich CA, using excitation wavelength of 310 nm and emission wavelength of 420 nm, the fluorescence in 250 mM KOH was 1829 ± 35 RFU and the S/B was 3.42 ± 0.10. At flanking concentrations, both RFU and S/B declined. Thus, in 100 mM KOH, the RFU and S/B were 1231 ± 20 and 2.23 ± 0.06, respectively and in 500 mM KOH, these values were 1165 ± 30 RFU and S/B of 2.10 ± 0.10, respectively. Since the signal strengths were comparable in 100 mM NaOH or 250 mM KOH, either base could be used. These results are in accordance with our fluorescence data of *p*-cresol [8] and hinted at similar ionization processes for both fluorophores. Furthermore, 100 mM NaOH or 250 mM KOH was also optimal for monitoring the fluorescence changes following laccase- or peroxidase-oxidation of CA.

### 2.5. Excitation and Emission Spectra of Laccase-Oxidized CA

**Scheme 1:**
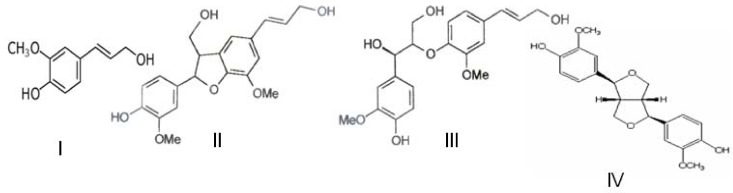
Chemical structures of CA and its main oxidation products. The various compounds shown are: coniferyl alcohol or CA (I), β-5 dehydrodimer or dehydrodiconiferyl alcohol (II), β-O-4 dehydrodimer or guaiacylglycerol β-O-4 coniferyl alchol ether (III) and β-β-dehydrodimer or pinoresinol (IV). It should be noted that compound III occurs in two different diastereoisomeric configurations of *erythro*-guaiacylglycerol-β-coniferyl ether and *threo*-guaiacylglycerol-β-coniferyl ether. In addition to these compounds, the so called dehydrogenation polymers (DHPs) and oligomers might also be formed depending upon the catalytic conditions such as the type and amount of enzyme (laccase or peroxidase) used, the duration of catalysis, reaction temperature, the concentration of the substrate, and other conditions.

Laccase oxidation diminished the fluorescence of CA with the magnitude of diminishment increasing between 0.01 to 0.25 μg/mL concentrations of laccase as seen in both the excitation and emission sweeps (Figure 4). However, the excitation and emission profiles of oxidized CA reactions were identical to the corresponding profiles of the unmodified CA (Figure 3). This indicated that we were monitoring the fluorescence of CA during catalysis and not the fluorescence of its oxidation products. Fluorescence decrease following oxidation might reflect a transformation of the CA into non-fluorescent products. This will result in lower levels of residual unmodified CA being present in the reaction mixtures, leading to diminished fluorescence but retaining the same excitation and emission profiles of unmodified CA. The data do not exclude a quench effect of the oxidation products on CA's fluorescence. Whether CA oxidation products (Scheme 1) are fluorescent or otherwise quench the fluorescence of unmodified CA is a topic for future work. This involves isolating the products, verifying structure and purity and testing each product for fluorescence and/or quench properties. This is beyond the scope of our current work where we are limiting to the demonstration of the hitherto unrecognized fluorescence properties of CA. 

### 2.6. Oxidation Kinetics of CA

**Figure 5:**
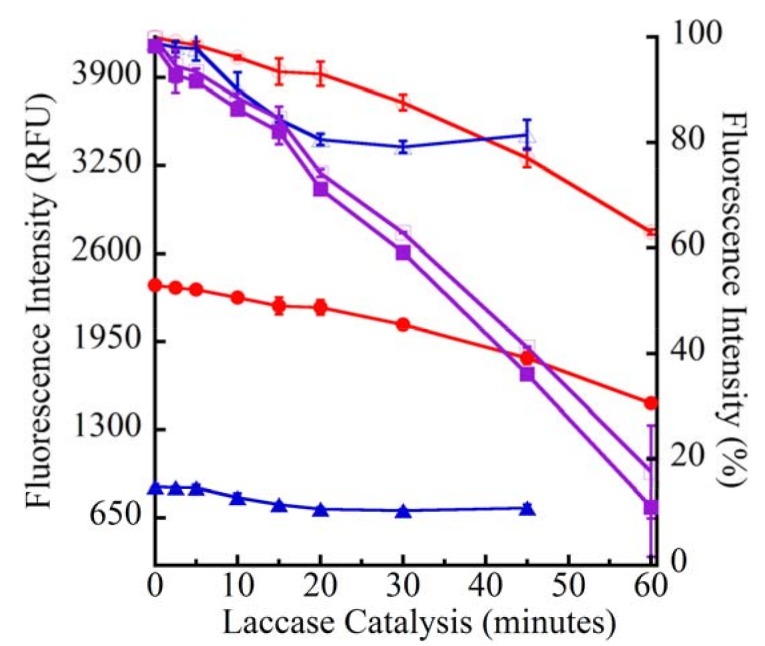
Laccase *pseudo*-kinetic oxidation of CA. Laccase (0.1 μg/mL) was used to oxidize 500 μM CA in pH 4.5 buffer in 96-well UV-transparent microplate. Identical reaction sets were stopped at the indicated time points using base (100 mM NaOH). The CA samples were then excited using 290 nm wavelength and the emission was monitored at 416 nm. The CA samples analyzed were purchased from Alfa Aesar (open and closed triangles, blue tracings), Acros Organics (open and closed circles, red tracings) and Sigma-Aldrich (open and closed squares, purple tracings). Open symbols represent percent decrease in fluorescence intensity and closed symbols are fluorescence intensities (RFU).

Similar to laccase reactivity of CA samples monitored by absorption spectroscopy (Figure 1 and Figure 2), the reaction profiles tracked in a like manner when examined by fluorescence spectroscopy (Figure 5). Thus, CA from Alfa Aesar had the smallest decline in fluorescence intensity consequent to laccase catalysis and the velocity saturated within 30 min. On the other hand, laccase oxidation of CA from Acros Organics and Sigma-Aldrich proceeded more gradually. Sigma-Aldrich CA displayed the greatest linearity of velocity over time. The oxidation profiles of Sigma-Aldrich CA also tracked closely whether analyzed by fluorescence decline or percent oxidation (Figure 5). The percent oxidation at 60 min was the greatest with Sigma-Aldrich CA (82%) followed by Acros Organics (37%) and trailed by Alfa Aesar (19%). These percent oxidations were remarkably close to the values determined in the absorption assay (Figure 1B) especially with CA from Acros Organics and Alfa Aesar. The higher percent oxidation of Sigma-Aldrich CA in the fluorescence assay (Figure 5) might be due to its greater sensitivity particularly for the better performing substrate. Reactivity of CA from the three vendors could be rank ordered as Sigma-Aldrich > Acros Organics > Alfa Aesar. The same trend lines were observed when the reactions were interrogated using 310nm and 340 nm excitation wavelengths. Absorption and fluorescence spectroscopic data were consistent for all CA samples, thus validating our QA model. 

### 2.7. Reproducibility of CA Fluorescence

Excitation and emission profiles identical to those shown in Figure 3 and Figure 4 were generated using CA from all three vendors, when the measurements were carried out by two different investigators working in different locations and using different microplate fluorometers. The CA sample from Sigma-Aldrich exhibited the highest dose-response relationship compared to CA of Acros Organics and Alfa Aesar. For example, in 96-well UV-transparent microplate, 250 and 500 μM of CA from Alfa Aesar yielded fluorescence intensities of 572 ± 38 and 843 ± 26 RFU compared to 4875 RFU from 250 μM CA from Sigma-Aldrich; greater than 10-fold difference. The CA samples from Sigma-Aldrich, Acros Organics and Alfa Aesar all displayed the characteristic fluorescence decrease following oxidation by laccase. However, the magnitude of fluorescence diminishment was the highest and therefore the Δ RFU values the largest, with CA from Sigma-Aldrich. Since both RFUs and S/B were highest with CA from Sigma-Aldrich, we used this reagent for the remainder of the experiments. 

### 2.8. Laccase Dose-Response

**Figure 6:**
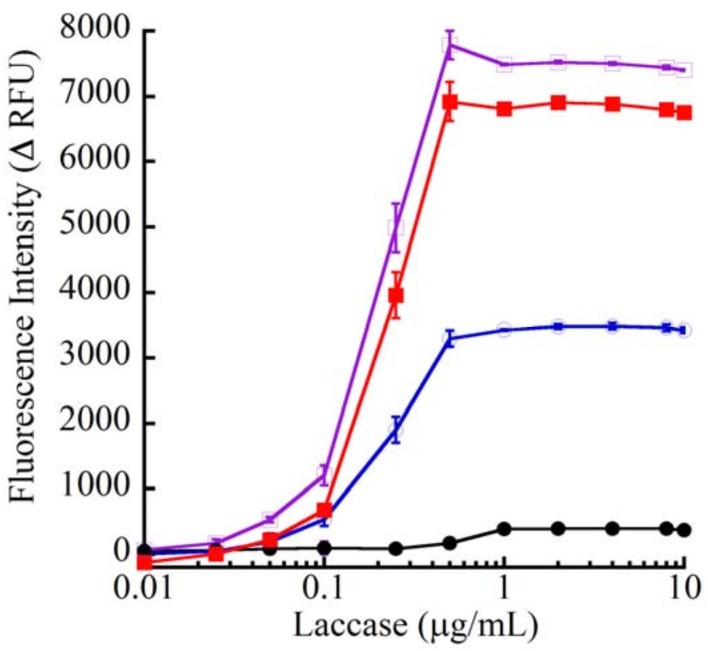
Laccase dose-response for CA oxidation. Increasing concentrations of laccase were used to oxidize 500 μM CA for 60 min. Reactions were carried out in 96-well white plate (100 μL/well) and stopped using 250 mM KOH (endpoint mode). Fluorescence was measured at 416 nm after exciting the samples using 290 nm (open squares, purple tracing), 310 nm (closed squares, red tracing), 340 nm (open circles, blue tracing) and 360 nm (closed circles, black tracing).

The dose-response profiles were similar when the Δ RFUs were tracked for 0.01 to 10 μg/mL enzyme and the reaction velocity saturated between ~0.5 to 10 μg/mL enzyme at all excitation wavelengths (Figure 6). However, the magnitude of the signal discrimination depended on the excitation wavelength with the Δ RFUs from 290 and 310 nm being similar and larger than the Δ RFUs obtained with 340 nm that in turn was greater than the Δ RFUs with 360 nm. The Δ RFUs became progressively larger with increasing concentrations of laccase due to the increasing fluorescence *diminishment* following CA oxidation. The use of base prior to fluorescence spectroscopy also stopped the enzymatic oxidation “instantaneously” due to laccase denaturation and by shifting drastically from the optimum pH for laccase [8]. Thus, laccase oxidation of CA could be monitored in *pseudo*-kinetic or endpoint mode by fluorescence spectroscopy (Figure 4, Figure 5 and Figure 6). The lag phase in the reaction velocity observed in Figure 6 might be due to the logarithmic increase in the concentration of the enzyme.

### 2.9. Kinetics of Laccase- and Peroxidase-Catalyzed CA Oxidation 

**Figure 7:**
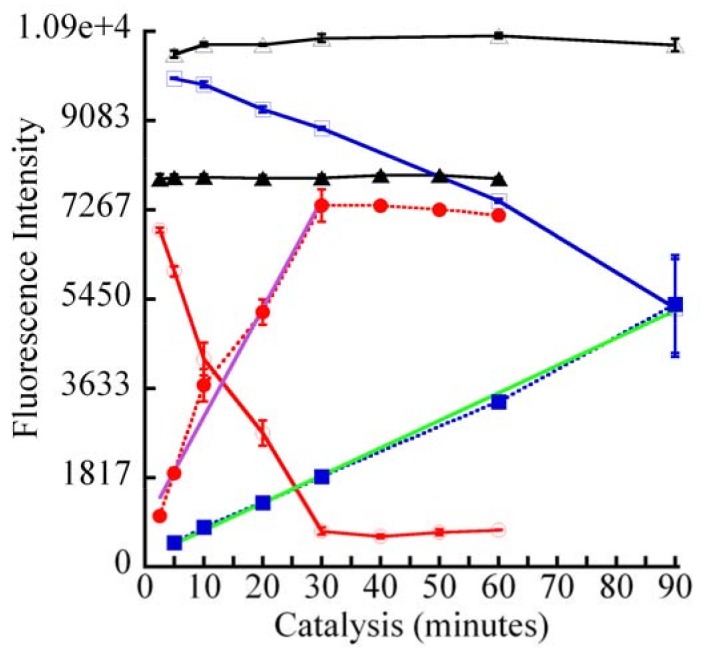
*Pseudo*-kinetics of laccase- and peroxidase-catalyzed oxidation of CA. Laccase (0.5 μg/mL) or peroxidase (0.1 μg/mL) was reacted with 250 μM CA in 96-well white plates for the indicated duration and then stopped with 250 mM KOH. Fluorescence was measured using λex of 290, 310 and 340 nm with λem of 416 nm. Due to the nearly identical kinetic profiles, only the data from λex of 290 nm is shown. The black tracings are “control” fluorescence from unmodified CA during peroxidase (closed triangles) or laccase (open triangles) catalysis. Closed circles (red dotted tracing) represent Δ RFU from peroxidase oxidized CA with linearity (*r^2^*) between 2.5 and 30 min of 0.978 (solid purple curve fit). Open circles (solid red tracing) are decreasing emission intensities over time following peroxidase catalysis. Open squares connected by the solid blue tracing represent fluorescence decline due to laccase oxidation of CA. Closed squares connected by dotted blue tracing are the upward trajectory of Δ RFU values from laccase-oxidized CA. Linearity (*r^2^*; solid green tracing) was 0.997.

Peroxidase is a major enzyme along with laccase influencing lignin biosynthesis and degradation [2,5]. In order to expand the fluorescence spectroscopic analyses of CA, we monitored the Δ RFU trajectories of laccase- and peroxidase-oxidized CA in *pseudo*-kinetic mode since progress curve is an indicator of enzyme-catalyzed reactions [13]. We also used these results to demonstrate our Δ RFU analyses in a graphical format (Figure 7). The peroxidase and laccase assays were done using different microplate readers located at two different test sites. The fluorescence intensity of CA in the absence of peroxidase (7930 ± 55 RFU; *n* = 24; <1% error) or laccase (10647 ± 131 RFU; *n* = 18; <2% error) underwent little change over the 60 to 90 min reaction period (Figure 7). Differences in the fluorescence intensities of the CA could be due to instrument variation at the two test locations. Parenthetically, this data provided the first hint of CA stability. Laccase or peroxidase oxidation of CA led to progressively diminishing fluorescence intensity over time as seen by the tracings with the negative slopes (Figure 7). On the other hand, the Δ RFUs had an upward trajectory. Laccase and peroxidase catalysis displayed first order reaction rate with linear trend lines between 2.5 and 30 min (peroxidase) or 60 min (laccase), indicating that the formation of oxidation products was proportional to the catalysis time. The rapid saturation of velocity at a lower concentration of peroxidase might indicate a greater reactivity of this enzyme towards CA relative to laccase (Figure 7). The data showed that laccase or peroxidase oxidation of CA could be detected within 5 min. 

Laccase is an important enzyme secreted by white rot *Basidomycetes* such as *Trametes versicolor* [2,4,5], the fungal source of our enzyme. The demonstration of physiologically-relevant substrates such as CA is useful for understanding the role of these phenoloxidative enzymes during delignification [2,4,5]. Finally, the data demonstrated that even though highly basic conditions were required for eliciting fluorescence from unmodified and oxidized CA, the assay was still useful for determining the oxidation kinetics through *pseudo*-kinetic analysis (Figure 7). Basification instantaneously stopped the catalysis at the desired time point and also facilitated maximum emission. With robotics and liquid handling systems, these steps can be integrated and automated for HTS [7]. The endpoint and *pseudo*-kinetic analyses conferred flexibility to our fluorescence assays of CA and its oxidation by laccase or peroxidase.

### 2.10. Dose-Response for Peroxidase Oxidation of CA

**Figure 8:**
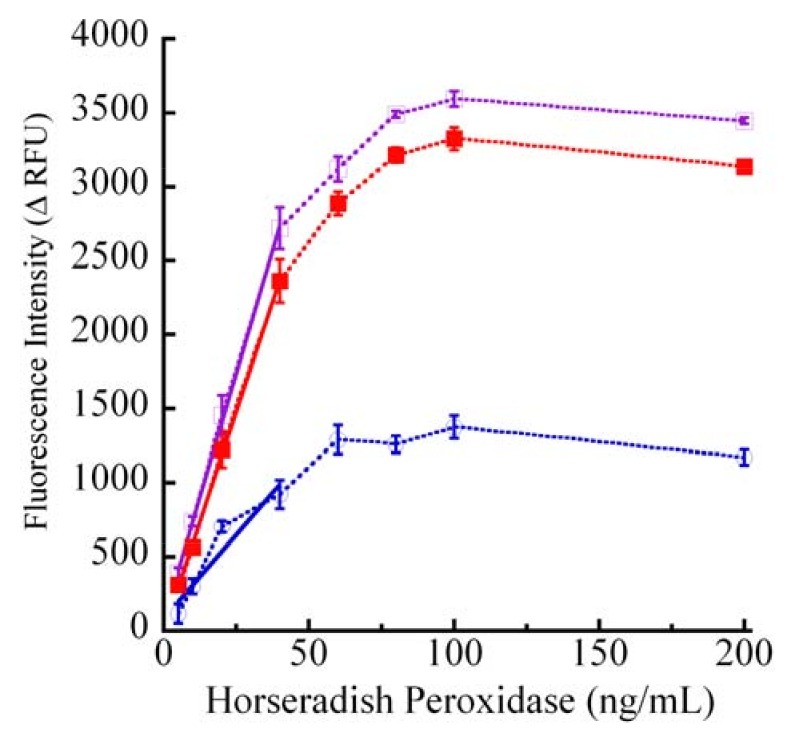
Peroxidase-catalyzed CA oxidation. Increasing concentrations of horseradish peroxidase were used to oxidize 250 μM CA in the presence of 250 μM H_2_O_2_ for 30 min using 96-well UV-transparent microplate. The reaction was stopped using 100 mM NaOH and the fluorescence was measured at 416 nm following excitation using 290 (open squares, purple), 310 (closed squares, red) and 340 nm (open circles, blue) wavelengths. Dotted tracings are interpolations and solid tracings are the linear curve fit (5 to 40 ng/mL peroxidase). Linearity (*r^2^*) values were 0.999 (290 nm, solid purple), 0.999 (310 nm, solid red) and 0.903 (340 nm, solid blue).

Similar to laccase, the magnitude of CA’s fluorescence decrease was related to peroxidase catalysis duration (Figure 7) or peroxidase concentration (Figure 8). The dose-response tracings showed a linear (correlation of coefficient, *r^2^* ≥ 0.90) increase in reaction velocity at low concentrations (up to 50 ng/mL peroxidase) of enzyme followed by zero order at higher concentrations (50 to 200 ng/mL enzyme) at all excitation wavelengths examined (Figure 8). Depending on the reaction conditions, as little as 2.5 ng/mL peroxidase was sufficient to elicit a measurable Δ RFU. This further confirmed the fluorescence properties of CA. Since laccase and peroxidase oxidation diminished the fluorescence, both enzymes might be acting promiscuously upon CA. It is likely that the reaction products from both catalysts might be the same or similar, based upon their absorption and fluorescence responses [6].

### 2.11. High Throughput Screening (HTS) Optimization

A goal of our work was to establish HTS conditions for the analyses of biofuel-critical phytochemicals such as CA. The advantages of HTS assays are speed, sample conservation due to small reaction volumes, lower cost, less waste, shorter turnaround of data, automation for unattended, 24 × 7 operations, and the ability to screen hundreds of thousands of samples every day. Paired with bioinformatics, lignin compositional analyses can be rapidly connected to the best pre-treatment strategy for improving saccharification. 

The fluorescence emission profiles of CA were nearly identical when the reactions were carried out using UV-transparent or opaque white plates. Fluorescence intensity and S/B were approximately double with white plates due to light reflectance from the well walls. The reaction volumes could be varied from 25 μL (384-wells) to 300 μL (96-wells) for measuring unmodified CA as well as laccase- or peroxidase-oxidized CA. These data are useful for transitioning our assays to ultra-HTS (1536-wells) with robotics-aided liquid handling, guided by the design considerations described before [7]. One aspect of HTS is inhibitor evaluation of compound libraries. We therefore investigated halide inhibition of laccase oxidation of CA as a model for compound library screening [8]. 

**Figure 9:**
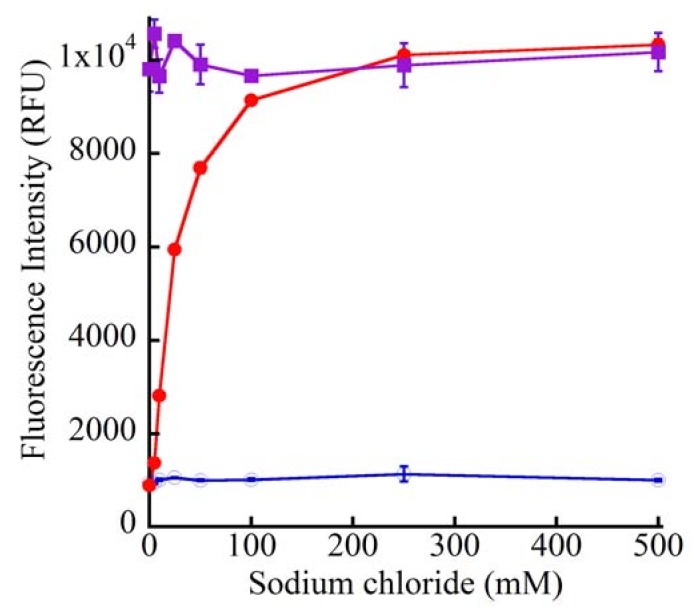
Halide inhibition of laccase-catalyzed CA oxidation. Indicated concentrations of NaCl were added either during the enzymatic reactions (closed circles, red tracing) or after stopping the reactions with 250 mM KOH (open circles, blue tracing). The effect of NaCl on the fluorescence of unmodified CA is shown by the purple tracing connecting the closed squares. Nearly identical profiles were generated by exciting the samples at 290, 310 and 340 nm and measuring the emission at 416 nm. The data shown in this figure were obtained by excitation of CA at 290 nm.

Halides bind to the copper atoms of fungal laccases which interrupts the electron shuttle and thereby inactivates the enzyme. Laccase inhibition profiles by halides are complex and calculation of the IC_50_ values might be adequate to document inhibition of laccase-catalyzed CA oxidation [14]. In agreement with our previous data of laccase inhibition of *p*-cresol oxidation [8], an IC_50_ of ~25 mM NaCl was calculated for the laccase inhibition of CA oxidation (Figure 9). With increasing concentrations of NaCl laccase is progressively inhibited, leading to the accumulation of unmodified CA resulting in increased fluorescence (Figure 9, red). Salt had no effect on the residual fluorescence of laccase-oxidized CA or unmodified CA fluorescence (Figure 9, blue and purple tracings). These data provide a basis for the HTS of laccase inhibitors using NaCl as an exemplar [8]. Reversibility of fluorescence diminishment due to laccase inhibition also affirmed the fluorescence properties of CA.

### 2.12. CA Fluorescence Sensitivity

Consistent with the data of Figure 3, the LOD and LOQ of Sigma-Aldrich CA were determined to be 1.8 and 6.9 μM, respectively (288 ppb and 1.2 ppm) (Table 1). We could detect and quantify 40 and 170 pmol respectively, of CA in 25 μL reactions using 384-well plate.

The LOD and LOQ were not different at excitation wavelengths of 290 and 310 nm. However, with 340 nm excitation the LOD was 2.5 μM CA. Fluorescence intensity was similar for 25 and 50 μL reactions with excitation wavelength of 290 or 310 nm (Figure 10A). 

Although the emission intensities were less discriminating at these wavelengths for 25 and 50 μL reactions, and the molar concentration of CA were identical, the *amount* of CA was double in 50 μL reactions compared to 25 μL. With 340 nm excitation, the emission became less intense for the 25 μL reactions relative to the 50 μL reactions (Figure 10A and Figure 10B). All dose-response tracings were linear (*r^2^* > 0.95) over two orders of magnitude of CA concentrations (1 to 100 μM) and the signals reached a plateau thereafter (Figure 10A and Figure 10B). These calculations were reproducible when the CA was retested more than two weeks later. 

A final point to note is that the same set of CA samples after absorption measurements in base [6] could be used for fluorescence measurements offering assay simplicity, ease and flexibility. Additional assay simplification might be achieved by carrying out the enzymatic and/or non-enzymatic reactions in the desired well volume at pH 4.5 using UV-transparent clear plate. After interrogation of the samples by absorption spectroscopy [6], concentrated base can be added such that the volume change is <5%. Finally, the samples can be re-interrogated using endpoint absorption [6, present work], *pseudo*-kinetic and endpoint fluorescence spectroscopy (present work). Thus, a single set of samples could be interrogated using multimode spectroscopy under real-time kinetic, *pseudo*-kinetic and endpoint modes, under acidic or basic conditions.

### 2.13. CA Fluorescence Stability

**Figure 12:**
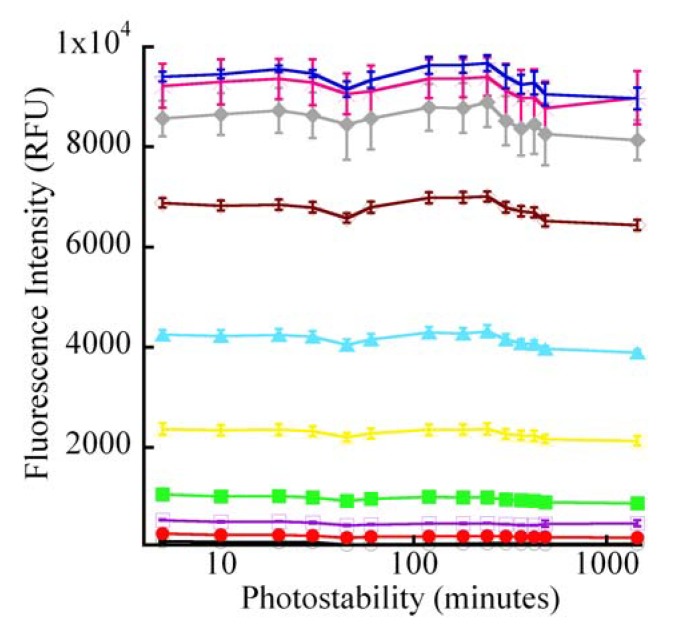
Time dependence of CA photostability. This figure shows the photostability of CA over time. All other reaction conditions were as described for Figure 11A and Figure 11B. The concentrations (μM) of CA are as follows: 1.0 (open circles, black), 2.5 (closed circles, red), 5 (open squares, purple), 10 (closed squares, green), 25 (open triangles, yellow), 50 (closed triangles, aqua), 100 (open diamonds, maroon), 250 (closed diamonds, grey), 500 (crosses, magenta) and 1000 (plusses, blue).

The certificate of analysis from three different vendors commented on the susceptibility of CA to air and light oxidation. We therefore studied the photostability, photodegradation and photobleaching of Sigma-Aldrich CA’s fluorescence. Since identical stability profiles were generated under all interrogation wavelengths and all the data were mutually consistent, only the data obtained with 310 nm excitation and 25 μL volume are presented in Figure 11 and Figure 12. The data demonstrated photostability of CA over three orders of concentrations (1 μM to 1000 μM) for 24 hours at ~25 ºC (Figure 11A and Figure 11B). 

Photostability was also observed over 14 cycles of excitation and emission at each of the 3 different excitation wavelengths, resulting in a total of 42 cycles, further documenting photostability. The unwavering intensity profiles over time and several concentrations of CA are evident from the tracings being nearly parallel to the abscissa (Figure 12) and the overlapping tracings in Figure 11A and Figure 11B. We conclude that the fluorescence properties of CA were stable at least over the duration of the HTS assays. Analyses in 384-well plate demonstrated the HTS capabilities and also corroborated the sensitivity data of Figure 10A and Figure 10B.

## 3. Experimental 

### 3.1. Materials

Coniferyl alcohol (98%–99% pure according to the certificates of analysis) was purchased from Sigma-Aldrich (Saint Louis, MO), Alfa Aesar (Ward Hill, MA) and Acros Organics (Geel, Belgium) and used as received. According to Sigma-Aldrich’s Fourier transform (FT) infrared (IR) Raman spectroscopy, FT-nuclear magnetic resonance (FT-NMR) spectroscopy and FT-IR spectroscopy data and Acros Organics NMR data posted on the company websites conformed to the structure. Stock solutions of CA, *Trametes versicolor* laccase (Sigma-Aldrich) and streptavidin-conjugated horseradish peroxidase (Pierce Chemical, Rockford, IL) were diluted in citrate buffer, pH 4.5 (Fluka, purchased through Sigma-Aldrich) as described previously [6,8,9]. White 96- and 384-well microplates were from Perkin-Elmer (Waltham, MA). UV-transparent 96-well microplates were from Fisher Scientific (Pittsburgh, PA). Microplate-sealing tape was from Pierce. Citrate buffer, pH 4.5 was used for analyzing unmodified CA and laccase- or peroxidase-catalyzed CA oxidation. Basic pH reactions were done in 100 mM NaOH or 250 mM KOH (final pH after addition of base was 13.0 ± 0.1). 

### 3.2. Absorption Spectroscopy

Absorption measurements were taken at ~25 ºC as described previously [6,8]. The absorption of CA in pH 4.5 buffer or in base was measured using 96-well UV-transparent microplate or quartz cuvette. Absorption spectra were generated by measuring the absorption intensities (A.U.) over the wavelengths (λ) of 250 to 350 nm in 2 nm intervals using a microplate reader (Spectramax M2, Molecular Devices Sunnyvale, CA). 

### 3.3. Fluorescence Spectroscopy

Fluorescence intensity (relative fluorescence units, RFU) from basified mixtures of CA and laccase- or peroxidase-oxidized CA were measured at ~25 ºC using 96- or 384-well microplate as described previously [8,9]. Samples were mixed by pipetting the solutions up and down using a multi-channel pipettor and/or by using the “automix” function of the microplate reader. The excitation spectrum was generated by holding the emission wavelength constant and varying the excitation over the wavelength of the absorption spectrum. Emission spectrum was generated with a fixed excitation wavelength. Fluorescence measurements were taken using Molecular Device M2 microplate reader.

### 3.4. Enzymatic Oxidation

Laccase- or peroxidase-catalyzed oxidation of CA was carried out at ~25 ºC in citrate buffer, pH 4.5 using 96-well microplates [6,8,9]. Laccase oxidation was by mixing the enzyme and the substrate (CA) in buffer. Peroxidase oxidation involved mixing the enzyme and CA in buffer and initiating the catalysis by adding H_2_O_2_ (250 μM final). Alternately, CA and H_2_O_2_ were mixed and catalysis was initiated by adding the enzyme. Substrate, enzyme, buffer, base, peroxidase+250 μM H_2_O_2_ without CA and CA+250 μM H_2_O_2_ without peroxidase were used as negative controls. Normally, kinetic reactions are monitored in real time. However, since basification was required in order to detect fluorescence, CA oxidation was monitored *pseudo*-kinetically by stopping identical enzymatic reactions at pre-determined timed intervals using base or in endpoint mode after fixed time by basification [6]. During *pseudo*-kinetics, reaction wells were mixed intermittently using the “automix” function. The CA fluorescence and absorption decreased upon oxidation. The magnitude of decrease was a measure of catalysis [6].

### 3.5. Halide Inhibition 

Sodium chloride was added to 96-well white microplate at the start of 0.5 μg/mL laccase oxidation of 250 μM CA at ~25 ºC for 60 min or after terminating the reactions with base. The former set of samples demonstrated the inhibitory effects of halide ions on laccase oxidation of CA. The latter set showed the effects (if any) of ionic strength on the fluorescence of oxidized CA. As an additional control, the effects of ionic strength on the fluorescence of unmodified CA (250 μM) were also tested. Additional details are in [8].

### 3.6. Stability Study

Ten different concentrations of CA, spanning three orders of magnitude (1 to 1000 μM) were prepared in 100 mM NaOH and added in 25 or 50 μL volumes to 384-well white microplate. CA samples were interrogated over 24 hours at 14 timed intervals (5 to 1440 min) using three different excitation wavelengths (290, 310 and 340 nm). Emission was monitored at 416 nm. To minimize the evaporation, microplate sealing tape was used between measurements. To protect the samples from light, the plate was also covered with an aluminum foil between measurements. 

### 3.7. Data Analysis 

All reactions were carried out at least in triplicate. Results were calculated as average ± standard deviation. Linearity (correlation of coefficient, *r^2^*), limits of detection (LOD) and quantitation (LOQ) were calculated as described previously [6,8,9]. The magnitude of fluorescence decrease following oxidation was calculated by subtracting the RFU values of enzymatic reactions (signal, S) from the RFU values of CA (blank, B) (*i.e.*, B – S) and was designated as delta RFU (Δ RFU). Since the magnitude of fluorescence decrease increased with time or enzyme concentration, the Δ RFUs assumed a positive character. The S/B was also used to evaluate certain data.

## 4. Conclusions

The origin of lignin fluorescence is not clear due to structural complexities, role of solvent and other factors [15]. Lignin was reported to fluoresce at 360–395 nm after excitation over 240–340 nm wavelengths [16,17], whereas lignin model compounds fluoresced at 370–465 nm [18]. Environmental sampling for lignin involved excitation over the wavelengths of 250–440 nm and monitoring the emission between 300–600 nm [19]. It is important to note that we have not tested environmental samples for interference in our fluorescent assays of CA. Lignin-CA polymer and cell wall-bound ferulic acid were identified as major fluorophores over the 440–530 nm emission wavelengths [20,21,22]. Ferulic acid and CA are identical except for the carboxyl group in the former replacing the hydroxyl group of the latter. We believe that this report is the first comprehensive description of the fluorescence properties of CA and fluorescence diminishment consequent to laccase and peroxidase oxidation. We described the advantages of the CA absorption assays recently [6]. These advantages are also relevant to the CA fluorescent assays and can be used in the coupled assays for enzymes that utilize, generate or liberate CA. These include vanillyl alcohol oxidase or eugenol oxidase that convert eugenol to CA and β-glucosidase that releases CA from coniferin [6, and references cited therein]. We hope to eventually deploy an orthogonal, field-portable, multiplexed, HTS biosensor for the *in situ*, on-site, standoff interrogation of plant biomass monolignols using absorption [6], fluorescence [8,9] and electrochemical [9] techniques. Our studies might aid in the rational selection of pretreatment strategies based on lignin compositional analyses in order to improve saccharification through the reduction of biomass recalcitrance for the production of cost-effective biofuels [1,2,3].

## Figures and Tables

**Figure 1A:**
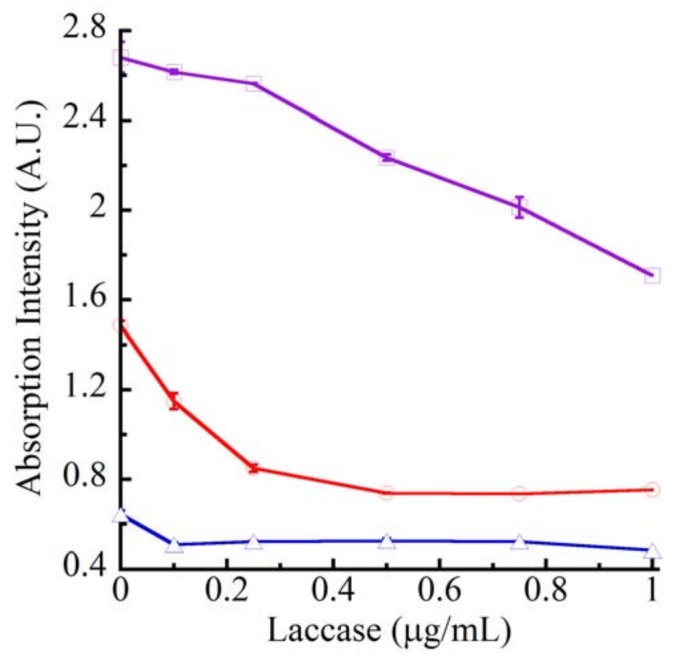
Laccase dose-response profiles for CA oxidation monitored by 260 nm absorption. Increasing concentrations of laccase were used to oxidize 500 μM CA in 100 μL of pH 4.5 buffer under real-time kinetic measurements using UV-transparent 96-well microplate. Decreases in CA absorbance were measured at 20 min for the samples purchased from Alfa Aesar (open triangles, blue tracing), Acros Organics (open circles, red tracing) and Sigma-Aldrich (open squares, purple tracing).

**Figure 1B:**
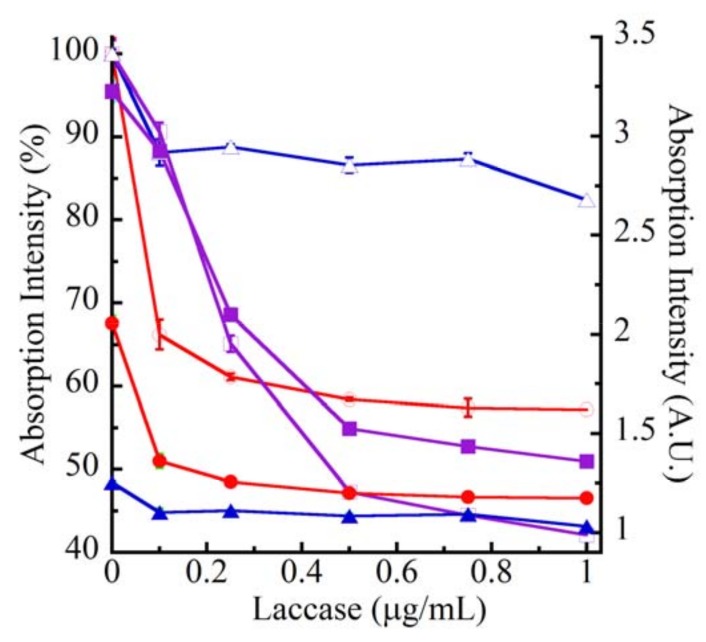
Laccase dose-response profiles for CA oxidation monitored by 290 nm absorption. Increasing concentrations of laccase were used to oxidize 500 μM CA in 100 μL of pH 4.5 buffer using UV-transparent 96-well microplate. At 60 min, the reactions were stopped through basification (100 mM NaOH final). Decreases in absorbance were measured from the CA samples purchased from Alfa Aesar (open and closed triangles, blue tracings), Acros Organics (open and closed circles, red tracings) and Sigma-Aldrich (open and closed squares, purple tracings). Open symbols represent normalized percent decreases in the absorption intensity and closed symbols are the actual absorption units (A.U.).

**Figure 10A:**
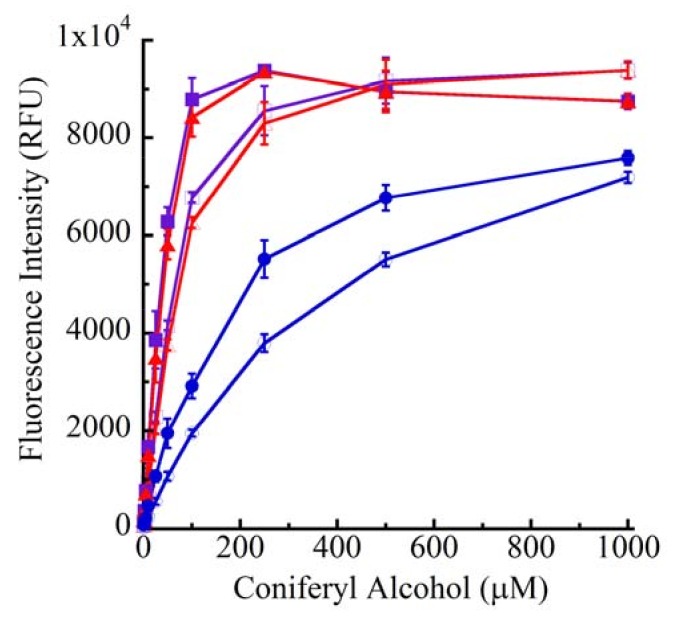
Sensitivity of CA. Increasing (1.0 to 1000 μM) concentrations of unmodified CA diluted in 100 mM NaOH were dispensed into a 384-well white plate as 25 or 50 μL solutions. Fluorescence from the samples was measured at 416 nm using the excitation wavelengths of 290 (purple tracings; open squares, 25 μL; closed squares, 50 μL), 310 (red tracings; open triangles, 25 μL; closed triangles, 50 μL) and 340 nm (blue tracings; open circles, 25 μL; closed circles, 50 μL).

**Figure 10B:**
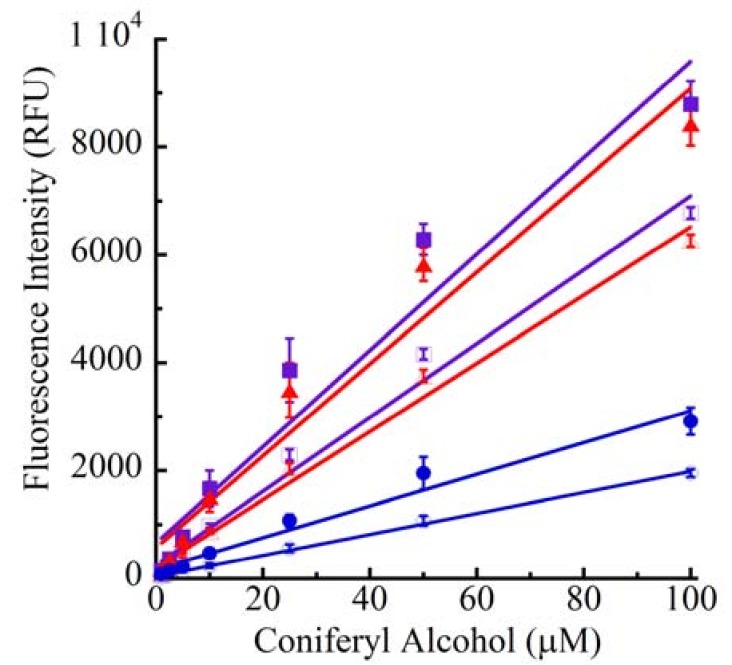
Linearity of CA Dose-Response. All conditions are described in Figure 10A. The *r^2^* values were: 0.984 (λex = 290 nm, 25 μL; open squares, purple); 0.945 (λex = 290 nm, 50 μL; closed squares, purple); 0.989 (λex = 310 nm, 25 μL; open triangles, red); 0.958 (λex = 310 nm, 50 μL; closed triangles, red); 0.997 (λex = 340 nm, 25 μL; open circles, blue) and 0.973 (λex = 340 nm, 50 μL; closed circles, blue).

**Figure 11A:**
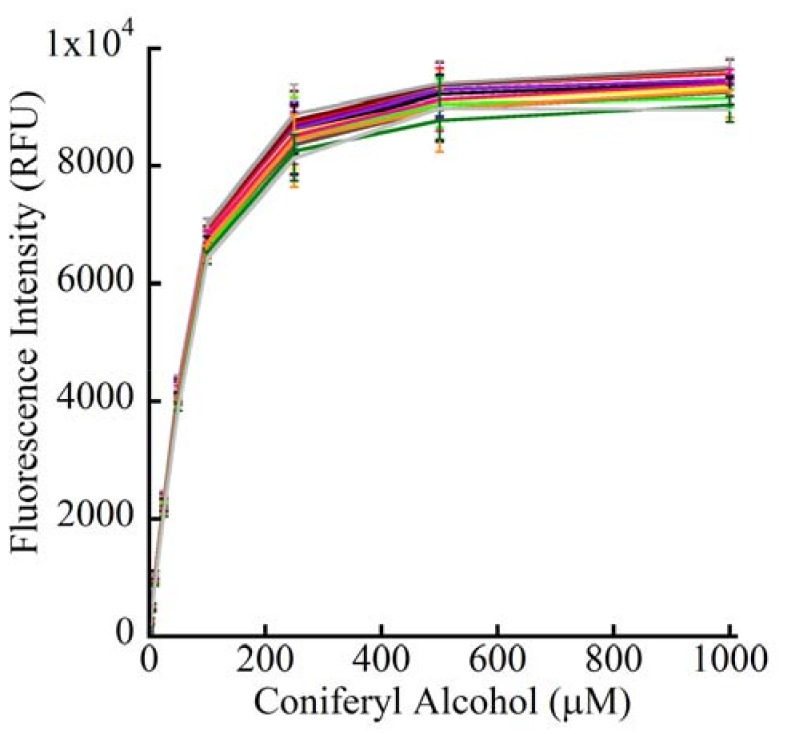
Concentration dependence of CA photostability. Increasing concentrations of CA were dispensed into 384-well microplate. The data from the 25 μL reactions (λex, 310 nm; λem = 416 nm) are shown. The tracings represent measurements taken at the following intervals (min): 5 (black), 10 (blue), 20 (red), 30 (purple), 45 (bright green), 60 (yellow), 120 (aqua), 180 (maroon), 240 (dark grey), 300 (magenta), 360 (brown), 420 (orange), 480 (dark green) and 1440 (light grey).

**Figure 11B:**
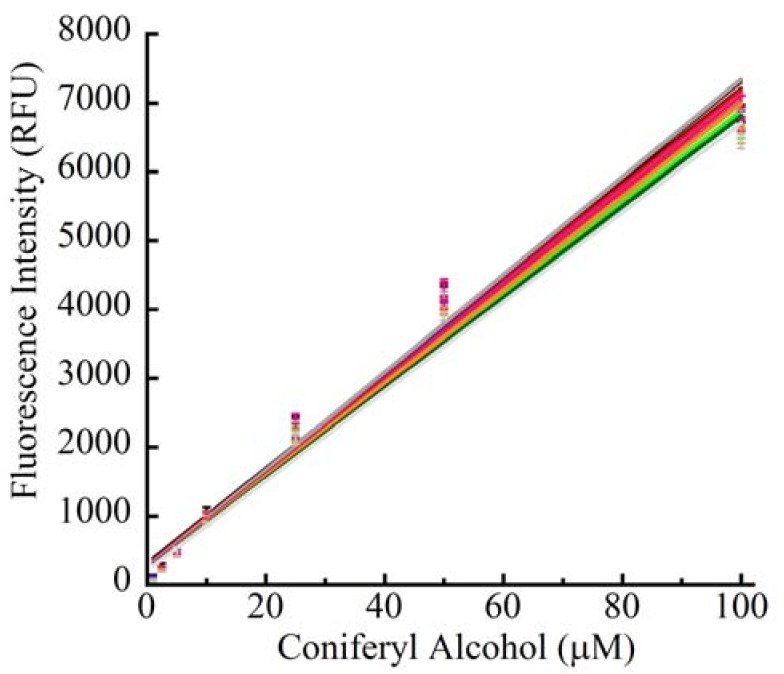
Linearity of CA photostability. This figure shows the negligible effects on linearity between 1.0 to 100 μM CA over the various interrogation times. The correlation of coefficient, *r^2^* was 0.985 ± 0.001 for *n* = 14; an error of <0.2% from CA samples subjected to 42 cycles of excitation/emission over a 24 h period. All other conditions and the colored tracings representing measurements between 5 and 1440 min are the same as those described for Figure 11A.

**Table 1:** Limits of detection (LOD) and quantitation (LOQ) of CA.*

Excitation wavelength (λ)	LOD (μM)	LOD (ppb)	LOQ (μM)	LOQ (ppm)	Linearity (*r^2^*)
290 nm	1.5	263	6.5	1.2	0.989
310 nm	1.5	259	7.7	1.4	0.970
340 nm	2.5	435	6.7	1.2	0.910

^*^ Fluorescence from CA solutions in 250 mM KOH was measured at 416 nm.
